# Online Detection of Peroxidase Using 3D Printing, Active Magnetic Mixing, and Spectra Analysis

**DOI:** 10.1155/2017/5031809

**Published:** 2017-04-24

**Authors:** Shanshan Bai, Chengqi Gan, Gaozhe Cai, Lei Wang, Mingyong Chen, Qingan Han, Jianhan Lin

**Affiliations:** ^1^Key Laboratory of Agricultural Information Acquisition Technology (Beijing), Ministry of Agriculture, China Agricultural University, Beijing, China; ^2^College of Veterinary Medicine, China Agricultural University, Beijing, China; ^3^Hebei Animal Disease Control Center, Shijiazhuang, China; ^4^Modern Precision Agriculture System Integration Research Key Laboratory of Ministry of Education, China Agricultural University, Beijing, China

## Abstract

A new method for online detection of peroxidase (POD) using 3D printing, active magnetic mixing, fluidic control, and optical detection was developed and demonstrated in this study. The proposed POD detection system consisted of a 3D printing and active magnetic mixing based fluidic chip for online catalytic reaction, an optical detector with a fluidic flow cell for quantitative determination of the final catalysate, and a single-chip microcontroller based controller for automatic control of two rotating magnetic fields and four precise peristaltic pumps. Horseradish peroxidase (HRP) was used as research model and a linear relationship between the absorbance at the characteristic wavelength of 450 nm and the concentration of HRP of 1/4–1/128 *μ*g mL^−1^ was obtained as *A*  =  0.257ln⁡(*C*) + 1.425 (*R*^2^  = 0.976). For the HRP spiked pork tests, the recoveries of HRP ranged from 93.5% to 110.4%, indicating that this proposed system was capable of detecting HRP in real samples. It has the potential to be extended for online detection of the activity of other enzymes and integration with ELISA method for biological and chemical analysis.

## 1. Introduction

Enzyme-linked immunosorbent assay (ELISA) has been widely used in the fields of clinical medicine, animal quarantine, food analysis, and so on [1–4]. Based on the antigen-antibody reaction, it often immobilizes the primary antibodies against the target on the solid phase to react with the targets in a sample. After washing to remove the sample background, the enzyme-labeled secondary antibodies are used to react the targets to form the sandwich complexes. When the redundant secondary antibodies are removed by washing, the substrate is added and catalyzed by the enzymes in the sandwich complexes, and the color of the substrate will change, which can be detected by an optical detector to determine the concentration of the target [5–7]. ELISA is often featured with high throughput, automatic operation, good specificity, and short detection time, but it has a relative high false positive ratio because cross-contamination and nonspecific adsorption often occur [8–10]. Therefore, it is important for ELISA or other enzymatic catalysis based assays to develop automatic detection methods of the enzymatic activity without cross-contamination.

Peroxidases (PODs) are a large family of enzymes, which are widely found in animals and plants, and can typically catalyze a reaction of the form:

(1) $$\begin{array}{c} {ROOR}^{\prime }+\overset{electron\;donor}{\overset{︷}{2e^{-}}}+2H^{+}\overset{Peroxidase}{\to }ROH+R^{\prime }OH \end{array}$$

For many of these PODs, the optimal substrate is hydrogen peroxide. However, hydrogen peroxide is often replaced with urea hydrogen peroxide (UHP) due to easy decomposition of hydrogen peroxide [11]. PODs, especially horseradish peroxidase (HRP), are used extensively in biochemical assays primarily for their ability to amplify a weak signal and thus increase the detection sensitivity of a target molecule [12]. Therefore, POD was used as research model in this study.

Lab-on-a-chip technology has shown its great potential to improve the analysis speed, reduce the analysis cost, and achieve the multiplex chemical and biological assays. In general, it relies on the use of microscale or nanoscale fluidic structure, micropump, and microvalve to perform the automatic or semiautomatic analysis procedures, including mixing, reaction, separation, and detection, on a miniaturized chip. Ibarlucea et al. reported a miniaturized lab-on-a-chip system for electrochemical and optical determination of biological analytes, which undergo enzyme-catalyzed reactions by integrating a biofunctionalized microfluidic mixer and a measurement chamber [13]. The lower detection limits for electrochemical and optical detection of glucose were 0.064 mM and 0.23 mM, respectively. Rodríguez-Ruiz et al. described a photonic lab-on-chip system with an enzymatically functionalized microfluidic reactor for monitoring the enzymatic catalytic reactions in continuous flow with a lower detection limit of 1.14 *μ*M for horseradish peroxidase [14]. Davidsson et al. developed a microfluidic flow injection system for quantitatively monitoring the dynamic production of glucose and ethanol using chemiluminescent biosensors with either coimmobilized glucose oxidase-horseradish peroxidase or alcohol oxidase-horseradish peroxidase, showing the possibilities and advantages of using a microfluidic system setup for cell-based assays [15]. Therefore, in this study we intended to use the lab-on-a-chip concept to develop an online detection method of the peroxidase activity.

## 2. Materials and Methods

### 2.1. Materials

Horseradish peroxidase and urea hydrogen peroxidase with the purity of ≥97% were purchased from Sigma (Saint Louis, MO, USA). 3,3′,5,5′-Tetramethylbenzidine (8.29 × 10^−4^ mol L^−1^) from Solarbio (Beijing, China) was used as the catalytic substrate. Sulfuric acid with the purity of ≥98% from Sinopharm (Beijing, China) was diluted to 0.2 mol L^−1^ as the catalytic terminator. Bovine serum albumin (BSA) from Solarbio was used to block the fluidic chip and the flow cell. Phosphate buffered saline (PBS, 10 mM, pH 7.4) was purchased from Sigma. Deionized water (18.2 MΩ cm) was produced by a Millipore Mill-Q system (A10, Bedford, MA, USA) and used for preparation of all the solutions.

The Silicone elastomer kit (SYLGARD 184) was purchased from Dow Corning (Auburn, MI, USA) for fabricating the poly(dimethoxy)silane (PDMS) channels. The printing material (Vero White plus RGD835, Stratasys, Eden Prairie, MN, USA) was used with the Objet24 3D printer to fabricate the molds of the channels. Polymethylmethacrylate (PMMA) was obtained from Haowei Plastic Industry (Shanghai, China) for bonding with the PDMS channels to fabricate the fluidic chip.

### 2.2. Setup of the Online POD Detection System

The proposed online POD detection system is based on 3D printing, active magnetic mixing, fluidic control, and optical detection. The principle of this proposed system can be expressed as follows:

(2) Urea  Hydrogen  Peroxide→PeroxidaseAtomic  Oxygen

(3) Oxygen+TMB substrate⟶Blue  Catalysate unstable

(4) Blue  Catalysate+H2SO4⟶Yellow  Catalysate stable

(5) Yellow  Catalysate→Optical  detectionPOD  Concentration

**Figure 1:**
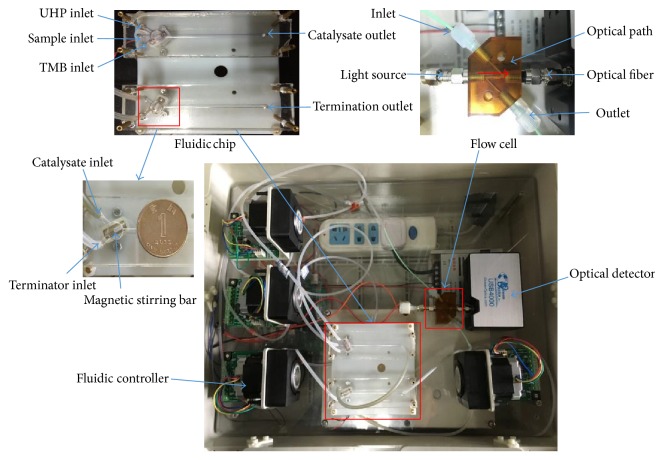
The proposed online peroxidase detection system, including a fluidic chip with two magnetic stirrers for online catalytic reaction, an optical detector with a flow cell for absorption measurement, and a fluidic controller for automatic operations of the fluids.

As shown in Figure 1, the sample containing PODs, UHP, and TMB was simultaneously pumped into the catalyzing channel of the fluidic chip. They were online mixed by a magnetic stirrer, which was embedded inside the channel and controlled by a rotating magnetic field. Inside the channel, UHP was catalyzed by the PODs to produce the atomic oxygen (see (2)), which could oxidize TMB to produce an unstable blue intermediate catalysate (see (3)). Then, the intermediate catalysate was simultaneously pumped with the sulfuric acid into the terminating channel of the fluidic chip to terminate the catalytic reaction and a stable yellow final terminated catalysate was obtained (see (4)), which was finally pumped into a flow cell for optical detection at a characteristic wavelength to determine the concentration of the PODs in the sample (see (5)).

### 2.3. Design and Fabrication of the Fluidic Chip

**Figure 2:**
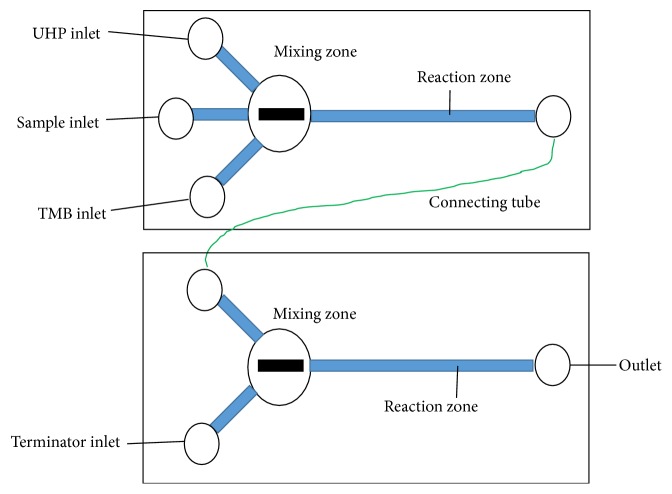
Schematic diagram of the fluidic chip.

The fluidic chip with active and passive mixing is the most important component for the development of the proposed online POD detection system [16–19]. It consisted of two parts: (1) the catalyzing channel for rapid and efficient mixing of the sample with the substrates to perform the online enzymatic catalysis reaction and obtain the blue intermediate catalysate and (2) the terminating channel for rapid and efficient mixing of the catalysate with the terminator (H_2_SO_4_) to perform the online terminating reaction and obtain the yellow final catalysate. As shown in Figure 2, the catalyzing channel includes three inlets for injecting the sample containing PODs, UHP, and TMB, respectively, a mixing zone (8 mm in diameter and 3 mm in height) with a magnetic stirrer (2 mm in diameter and 5 mm in length) for online mixing of these three solutions, a reaction zone (1.8 mm in width, 1 mm in height, and 60 mm in length) for efficiently performing catalytic reaction, and an outlet for flowing out the blue intermediate catalysate. The terminating channel includes two inlets for injecting the intermediate catalysate and the terminator, respectively, the same mixing zone and the same reaction zone with the catalyzing channel, and an outlet for flowing out the stable yellow final catalysate for optical detection. The outlet of the catalyzing channel was connected with either inlet of the terminating channel with a tubing.

The fluidic chip was fabricated using 3D printing and replica molding [20–25]. First, the 3D drawings of the fluidic chip were drafted using the Solidworks software in  .stl format. Then, the molds of the chip were printed out using the 3D printer (Objet24, Stratasys, Eden Prairie, MN, USA) with an accuracy of 0.1 mm in *x*-axis and *y*-axis and 28 *μ*m in *z*-axis, followed by casting the degassed PDMS (silicone : curing agent = 8 : 1) at 60°C for 8 h. After the patterned PDMS channels were removed from the master molds, the channels and the flat PMMA sheets with the thickness of 2 mm were oxygen plasma treated using a Plasma Cleaner (PDC-32G-2, HARRICK, NY, USA) for 1 min, then bonded together, and finally placed at 65°C for 2 h to fabricate the fluidic chips. Prior to the plasma treatment, the PMMA sheets were placed in isopropanol to clean the surface for 10 min using an ultrasonic cleaner, followed by washing with deionized water and drying with nitrogen flow, and then immersed in aminopropyltriethoxysilane (5%) at 65°C for 2 min, followed by washing with deionized water and drying with nitrogen flow.

### 2.4. Automatic Control of the Flow and the Magnetic Mixing

The control strategy of the flow and the magnetic mixing is another key to achieve automatic operations of the online POD detection with less cross-contaminations. Both the flow of the solutions and the operation of the magnetic fields were automatically controlled by an electronic controller, which was developed using a single-chip microcontroller STC89C52 with four precise peristaltic pumps (T60-S2&WX10-14-A, LongerPump, Baoding, Hebei, China) with a flowrate range of 0–24 mL min^−1^, and two DC motors (RF-310TA-11400, Mabuchi Motor, Dongguan, Guangdong, China) with a working voltage range of 3–5 V, respectively.

For the online detection of the PODs or the control, the logic of the controller was shown in Table 1. First, immediately after the two DC motors were used to rotate two NdFeB Grade-N48 column magnets with a diameter of 5 mm, a length of 12 mm, and a strength of 0.12 T, respectively, and keep the two magnetic stirrers in the channels rotating continuously (at time *t*_1_), three peristaltic pumps were used to simultaneously inject the same volume of the sample, UHP and TMB into the catalyzing channel at the same flowrate (at time *t*_2_). Then, another peristaltic pump was used to inject the terminator into the terminating channel (at time *t*_3_), while the intermediate catalysate started to flow into the terminating channel. When the sample was completely injected into the fluidic chip, the pumps for the injection of UHP and TMB were stopped (at time *t*_4_). After all the intermediate catalysate was transported into the terminating channel, the pump for the terminator was stopped (at time *t*_5_). Finally, all the pumps and the motors were stopped when the final catalysate was filled in the flow cell (at time *t*_6_).

For the blocking of the fluidic chip and the flow cell, 1% BSA solution was continuously injected from the sample inlet to fill in the fluidic chip and incubate for 15 min. Then, the deionized water was continuously injected from the sample inlet at the flow rate of 1.25 mL min^−1^ for 5 min, which was also used for washing the fluidic chip and the flow cell immediate after one test.

### 2.5. Optical Detection of the Final Catalysate

The optical detection of the final catalysate has a close relation with the sensitivity of this proposed POD detection system [26, 27]. The optical system consisted of three parts: (1) the optical detector (USB 4000, Ocean Optics, Dunedin, FL, USA) for collecting the absorption spectra of the final catalysate, (2) the LED light source with a power of 1 W for providing the incident light, and (3) the Z-type brown flow cell working as both the flow channel and the optical channel. The flow cell was connected with the detector and the light source using two SMA905 fiber connectors and with the fluidic chip and the waste container using two P-630 connectors (IDEX Health & Science, IL, USA). The wavelength of the light source was determined based on the maximum change of the spectra for different concentrations of PODs with the optimal wavelength of 450 nm.

The amount of the final catalysate is determined by measuring its absorbance at the characteristic wavelength of 450 nm. The relationship between the concentration and the absorbance can be described by Lambert-Beer law.

(6) $$\begin{array}{c} A=\varepsilon cd, \end{array}$$

where *A* is the absorbance; *ε* is the absorption coefficient; *c* is the concentration; *d* is the optical depth. In this study, the optical depth is fixed to be the length of the flow channel; thus the absorbance is proportional to the concentration of the final catalysate.

### 2.6. Online Detection of the Peroxidases

**Figure 3:**
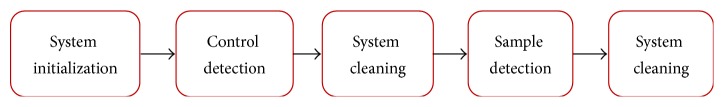
The procedure for online detection of the PODs.

The general procedure for online detection of the PODs is illustrated in Figure 3. First, the proposed POD detection system was blocked with 1% BSA for 15 min and washed with the deionized water for 5 min. Then, after the magnetic fields were turned on and rotated at 3000 rpm, 200 *μ*L of the sample containing PODs, UHP, and TMB was simultaneously injected into the fluidic chip at the same flowrate of 0.15 mL min^−1^. Finally, the final catalysate was detected by the optical detector and the absorbance at the characteristic wavelength of 450 nm was calculated to determine the concentration of the PODs.

For the POD spiked real sample tests, the pork provided by the Animal Disease Control Center of Hebei Province was used as real sample model and stored at −20°C prior to tests. The preparation of the pork sample was slightly modified based on the conventional sample preparation procedures. First, ~25 g of the pork sample was washed with the deionized water and grinded in a clean meat blender for 5 s after the adipose and connective tissues were removed. Then, 5 g of the pork was obtained and mixed with 45 mL of the deionized water at 15 rpm for 5 min using a rotator (MX-RL-E, SCILOGEX, CT, USA). After standing for 5 min, ~5 mL of the supernatant was filtered with a disposable injection filter (0.45 *μ*m) to remove the pork residues at a flow rate of 1 mL min^−1^, and the pork infusion sample was prepared and stored at 4°C for tests.

## 3. Results and Discussions

### 3.1. Selection of the Characteristic Wavelength

**Figure 4:**
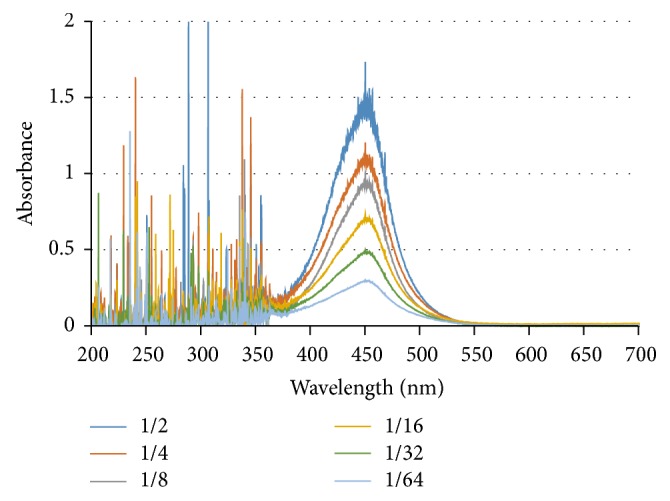
The spectra of the catalysate at different concentrations from 1/2 to 1/64 *μ*g mL^−1^.

The characteristic wavelength with the maximum absorbance of the final catalysate is important for the development of this proposed system. To find the characteristic wavelength, the pure HRP samples at different concentrations from 1/2 to 1/64 *μ*g mL^−1^ were used. 200 *μ*L of the HRP was mixed with 200 *μ*L of UHP (0.1 M) and TMB (8.29 × 10^−4 ^mol L^−1^) in a centrifuge tube at 15 rpm for 30 s to obtain the blue intermediate catalysate. Immediately after the mixing procedure, 100 *μ*L of H_2_SO_4_ with the concentration of 0.2 M was added to the catalysate and mixed at 15 rpm for 30 s to terminate the catalytic reaction. Finally, the final catalysate was placed in a cuvette and the spectra at the wavelength of 200 nm to 780 nm were collected using the USB4000 spectrophotometer. As shown in Figure 4, the spectra of the HRP at the higher concentrations have a higher absorbance because more yellow final catalysate was produced with deeper color. Besides, the maximum absorbance of the HRP occurs at the wavelength of ~450 nm and the characteristic wavelength was selected as 450 nm.

### 3.2. Optimization of the Flowrate

**Figure 5:**
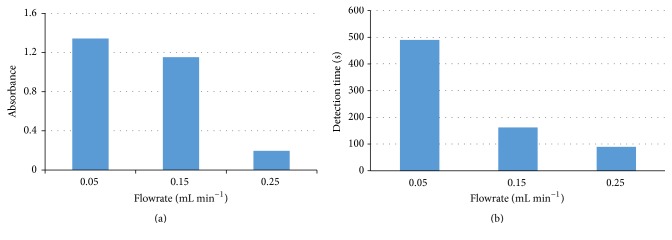
Comparison of the absorbance and the detection time at different flowrates.

The flowrate of injecting the sample, UHP, and TMB is a key factor to the sensitivity of this proposed system since it has a great impact on the catalytic reaction efficiency. Besides, it also can influence the detection time. To obtain the optimal flowrate, three pure HRP samples at the same concentration of 0.125 *μ*g mL^−1^ were detected at different flowrates (0.05, 0.15, and 0.25 mL min^−1^). 200 *μ*L of the HRP, UHP, and TMB was injected into the fluidic chip to sequentially obtain the blue intermediate catalysate and the yellow final catalysate. The spectra of the final catalysate were collected and the absorbance at the characteristic wavelength of 450 nm was used to optimize the flowrate. As shown in Figure 5, the absorbance decreases from 1.34 to 0.20 and the detection time also decreases from 490 s to 90 s, while the flowrate changes from 0.05 mL min^−1^ to 0.25 mL min^−1^. This indicated that when the higher flowrate was used, less amount of the final catalysate was produced in the reaction zone due to the shorter reaction time. Considering the absorbance and the detection time together, the optimal flow rate was 0.15 mL min^−1^.

### 3.3. Calibration Model of This Proposed System

**Figure 6:**
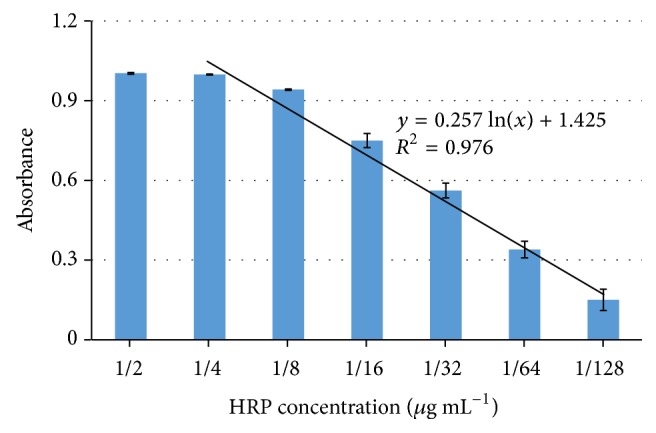
The relationship between the absorbance and the concentration.

The calibration curve is the basis for this proposed system to quantitatively determine the concentration of the PODs. Under the optimal conditions, triplicate tests of the pure HRP samples at different concentrations of 1/2 *μ*g mL^−1^ to 1/128 *μ*g mL^−1^ were conducted using this proposed system. As shown in Figure 6, a linear relationship between the absorbance (*A*) at the characteristic wavelength of 450 nm and the concentration (*C*) of HRP from 1/4 *μ*g mL^−1^ to 1/128 *μ*g mL^−1^ was obtained and could be expressed by *A* = 0.257ln⁡(*C*) + 1.425  (*R*^2^ = 0.976).

To obtain the lower detection limit of this proposed system, ten negative control samples were tested using this proposed system with an average absorbance value of 0.130 ± 0.034. The lower detection limit was determined by three times of signal to noise ratio and was calculated as 0.01 *μ*g mL^−1^. Besides, the repeatability of this proposed system was evaluated by conducting 5 parallel tests on the HRP samples at the concentration of 1/16 *μ*g mL^−1^. The mean absorbance at the characteristic wavelength was 0.75 ± 0.03; that is, the relative standard deviation was 4.0%, indicating that this proposed system had a good repeatability.

### 3.4. Comparison of This Proposed System with the Manual Method

**Figure 7:**
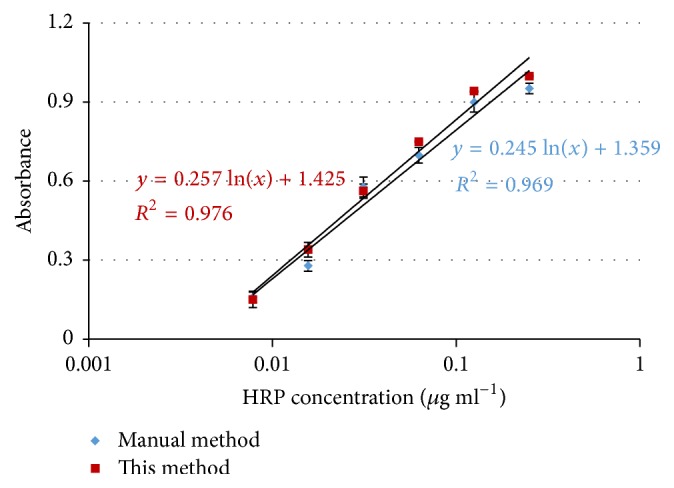
The comparison of this proposed automatic system with the manual method.

To compare this proposed system with the previously reported manual POD detection method, the pure HRP samples at the concentrations of 1/128 *μ*g mL^−1^ to 1/4 *μ*g mL^−1^ were prepared and detected using both this proposed automatic system and the manual method. For the manual detection, the same volume (300 *μ*L) of the HRP, TMB, and UHP was mixed at 15 rpm for 30 s. Then, 100 *μ*L of 0.2 M H_2_SO_4_ was added to the intermediate catalysate and mixed at 15 rpm for 10 s. Finally, the absorbance of the final catalysate at the characteristic wavelength of 450 nm was measured to calculate the amount of the HRP. As shown in Figure 7, the absorbance values of the final catalysate for different concentrations of the HRP using the manual method are almost consistent with those using this proposed system, and the sensitivity and repeatability of this proposed automatic system are slightly higher than those of the manual method. This is probably due to the following reasons: (1) this proposed system performing the automatic operations in the whole procedure owns a better consistency, resulting in a better repeatability and a higher signal to noise ratio; and (2) this proposed system has a higher efficient catalytic reaction in the fluidic chip, resulting in a better sensitivity. The detection time of this proposed system is 5 min for each test, which is a little longer than the manual method (~3 min). However, this proposed system was almost automatic and did not need intensive labor and well-trained technician, but the manual method depended on the proficiency of the technician, which greatly limited its practical application.

### 3.5. Detection of the HRP Spiked Pork Sample

To evaluate the applicability of this proposed POD detection system for rapid detection of the peroxidases in real sample, the HRP at the concentrations of 1/8 *μ*g mL^−1^ to 1/32 *μ*g mL^−1^ were added to the same pork infusion sample to prepare the HRP spiked pork infusion samples. The pork infusion sample was used as negative control, and the pure HRP at the same concentrations were used for comparison. Then, three parallel tests of the pork infusion samples were conducted using this proposed system. The recovery (*R*) of HRP was calculated by

(7) $$\begin{array}{c} R=\frac{C_{s}-C_{h}}{C_{a}}\times 100\%, \end{array}$$

where *C*_*a*_, *C*_*h*_, and *C*_*s*_ are the concentration of the pure HRP added to the pork sample, the concentration of the pork sample, and the concentration of the spiked sample measured using this proposed system, respectively. The absorbance of the control (the pork) was measured to be 0.35, indicating that the HRP concentration of this pork (*C*_*h*_) was 0.015 *μ*g mL^−1^. As shown in Table 2, the recoveries of HRP at different concentrations range from 93.5% to 110.4%, indicating that this proposed system was applicable for the detection of the PODs in real samples.

To the best of our knowledge, no literature has reported the use of lab-on-a-chip technology for the screening of the peroxidases. This proposed online POD detection system was demonstrated to be able to achieve automatic operations in the rapid detection of PODs, avoid cross-contamination, and reduce the requirements on the skilled technician and the facilities.

## 4. Conclusions

In summary, we have developed and demonstrated an online POD detection system using 3D printing, active magnetic mixing, fluidic control, and spectra analysis. The proposed POD detection system has a good linear range of 1/128 *μ*g mL^−1^ to 1/4 *μ*g mL^−1^ with a lower detection limit of 0.01 *μ*g mL^−1^. The detection time for each test is 5 min and could potentially be shortened in a smaller fluidic chip to enhance the reaction efficiency. This proposed system could achieve automatic operations in the detection of the PODs and greatly reduce the requirements on the skilled technician and the facilities. It has the potential to be extended for online detection of the activity of other enzymes and integration with ELISA method for biological and chemical analysis.

## Figures and Tables

**Table 1:** The control logic for the online detection.

Time	*t*_0_	*t*_1_	*t*_2_	*t*_3_	*t*_4_	*t*_5_	*t*_6_
UHP	0	0	1	1	0	0	0
Sample	0	0	1	1	1	1	0
TMB	0	0	1	1	0	0	0
Terminator	0	0	0	1	1	0	0
Motor 1	0	1	1	1	1	1	0
Motor 2	0	1	1	1	1	1	0

1: on; 0: off.

**Table 2:** Recovery of the HRP in the pork sample.

Conc. of added pure HRP (*C*_*a*_, *μ*g mL^−1^)	Absorbance of HRP spiked sample	Conc. of HRP spiked sample (*C*_*s*_, *μ*g mL^−1^)	Recovery (%)
0.125	0.94	0.153	110.4%
0.063	0.78	0.081	104.7%
0.031	0.62	0.044	93.5%

## References

[1] Westman K. W. A., Selga D., Bygren P. (1998). Clinical evaluation of a capture ELISA for detection of proteinase-3 antineutrophil cytoplasmic antibody. *Kidney International*.

[2] Yang J., Dai X., Chen H. (2016). Development of blocking ELISA for detection of antibodies against H9N2 avian influenza viruses. *Journal of Virological Methods*.

[3] Mao S., Ou X., Zhu D. (2016). Development and evaluation of indirect ELISAs for the detection of IgG, IgM and IgA1 against duck hepatitis A virus 1. *Journal of Virological Methods*.

[4] Zhu L., He J., Cao X., Huang K., Luo Y., Xu W. (2016). Development of a double-antibody sandwich ELISA for rapid detection of *Bacillus Cereus* in food. *Scientific Reports*.

[5] Van Weemen B. K., Schuurs A. H. W. M. (1971). Immunoassay using antigen-enzyme conjugates. *FEBS Letters*.

[6] Engvall E., Perlmann P. (1971). Enzyme-linked immunosorbent assay (ELISA) quantitative assay of immunoglobulin G. *Immunochemistry*.

[7] Clark M. F., Adams A. N. (1977). Characteristics of the microplate method of enzyme linked immunosorbent assay for the detection of plant viruses. *Journal of General Virology*.

[8] Huang S. Z., Du Y. Z., Zhang Y. H. (2006). Establishment and application of ELISA method for detecting *Salmonella* SPP. *Chinese Journal of Preventive Veterinary Medicine*.

[9] Holt P. S., Gast R. K., Greene C. R. (1995). Rapid detection of Salmonella enteritidis in pooled liquid egg samples using a magnetic bead-ELISA system. *Journal of Food Protection*.

[10] Kumar R., Surendran P. K., Thampuran N. (2008). Evaluation of culture, ELISA and PCR assays for the detection of Salmonella in seafood. *Letters in Applied Microbiology*.

[11] Ji L., Liu J., Qian C., Chen X. (2012). Advances in the application of urea-hydrogen peroxide to oxidation reactions. *Chinese Journal of Organic Chemistry*.

[12] Chattopadhyay K., Mazumdar S. (2000). Structural and conformational stability of horseradish peroxidase: effect of temperature and pH. *Biochemistry*.

[13] Ibarlucea B., Munoz-Berbel X., Ortiz P., Büttgenbach S., Fernández-Sánchez C., Llobera A. (2016). Self-validating lab-on-a-chip for monitoring enzyme-catalyzed biological reactions. *Sensors and Actuators B: Chemical*.

[14] Rodríguez-Ruiz I., Masvidal-Codina E., Ackermann T. N., Llobera A. (2015). Photonic lab-on-chip (PhLOC) for enzyme-catalyzed reactions in continuous flow. *Microfluidics and Nanofluidics*.

[15] Davidsson R., Johansson B., Passoth V., Bengtsson M., Laurell T., Emnéus J. (2004). Microfluidic biosensing systems Part II. Monitoring the dynamic production of glucose and ethanol from microchip-immobilised yeast cells using enzymatic chemiluminescent *μ*-biosensors. *Lab on a Chip—Miniaturisation for Chemistry and Biology*.

[16] Ansari M. A., Kim K.-Y., Anwar K., Kim S. M. (2012). Vortex micro T-mixer with non-aligned inputs. *Chemical Engineering Journal*.

[17] Chen J. J., Chen C. H., Shie S. R. Interfacial configurations and mixing performances of fluids in staggered curved-channel micromixers.

[18] Eickenberg B., Wittbracht F., Stohmann P. (2013). Continuous-flow particle guiding based on dipolar coupled magnetic superstructures in rotating magnetic fields. *Lab on a Chip*.

[19] Meijer H. E. H., Singh M. K., Kang T. G., Den Toonder J. M. J., Anderson P. D. (2009). Passive and active mixing in microfluidic devices. *Macromolecular Symposia*.

[20] McDonald J. C., Duffy D. C., Anderson J. R. (2000). Fabrication of microfluidic systems in poly(dimethylsiloxane). *Electrophoresis*.

[21] Alam A., Kim K.-Y. (2013). Mixing performance of a planar micromixer with circular chambers and crossing constriction channels. *Sensors and Actuators B: Chemical*.

[22] Ansari M. A., Kim K.-Y. (2010). Mixing performance of unbalanced split and recombine micomixers with circular and rhombic sub-channels. *Chemical Engineering Journal*.

[23] Jeon N. L., Chiu D. T., Wargo C. J. (2002). Design and fabrication of integrated passive valves and pumps for flexible polymer 3-dimensional microfluidic systems. *Biomedical Microdevices*.

[24] Neches R. Y., Flynn K. J., Zaman L., Tung E., Pudlo N. (2016). On the intrinsic sterility of 3D printing. *PeerJ*.

[25] Comina G., Suska A., Filippini D. (2014). PDMS lab-on-a-chip fabrication using 3D printed templates. *Lab on a Chip*.

[26] Sanz V., de Marcos S., Galbán J. (2007). A reagentless optical biosensor based on the intrinsic absorption properties of peroxidase. *Biosensors and Bioelectronics*.

[27] Rodriguez Maranon M. J., Mercier D., Van Huystee R. B., Stillman M. J. (1994). Analysis of the optical absorption and magnetic-circular-dichroism spectra of peanut peroxidase: electronic structure of a peroxidase with biochemical properties similar to those of horseradish peroxidase. *Biochemical Journal*.

